# Erratum: A cubesat centrifuge for long duration milligravity research

**DOI:** 10.1038/s41526-017-0025-9

**Published:** 2017-09-05

**Authors:** Erik Asphaug, Jekan Thangevelautham, Andrew Klesh, Aman Chandra, Ravi Nallapu, Laksh Raura, Mercedes Herreras-Martinez, Stephen Schwartz

**Affiliations:** 1 Arizona State University, Tempe, AZ USA; 2 Jet Propulsion Laboratory, Pasadena, CA USA

**Erratum to**: *npj Microgravity* (2017); doi:10.1038/s41526-017-0021-0; Published 05 June 2017

In the HTML version, Figs. 5 and 6 were duplications of other figures. These figures have now been removed in the HTML version of this Article.

